# Treatment Patterns of Pancreatic Neuroendocrine Tumor (pNET) Patients at Two Canadian Cancer Centres

**DOI:** 10.3390/curroncol32020086

**Published:** 2025-02-03

**Authors:** Gautham Nair, Morgan Black, Kathie Baer, Stephen Welch, David T. Laidley, Rachel Goodwin, Macyn Leung, William J. Phillips, Michael Vickers, Tim Asmis, Horia Marginean, Elena Tsvetkova

**Affiliations:** 1 Schulich School of Medicine and Dentistry, Western University, London, ON N6A 3K7, Canada; gnair7@uwo.ca; 2 London Health Sciences Centre, London, ON N6A 5W9, Canada; morgan.black@lhsc.on.ca (M.B.); kathie.baer@lhsc.on.ca (K.B.); stephen.welch@lhsc.on.ca (S.W.); david.laidley@lhsc.on.ca (D.T.L.); 3 The Ottawa Hospital (TOH), Ottawa, ON K1H 8L6, Canada; rgoodwin@toh.ca (R.G.); mvickers@toh.ca (M.V.); tasmis@toh.ca (T.A.); 4 Department of Medicine, University of Ottawa, Ottawa, ON K1N 6N5, Canada; 5 The Ottawa Hospital Research Institute (OHRI), Ottawa, ON K1H 8L6, Canada; macleung@ohri.ca (M.L.); hmarginean@ohri.ca (H.M.)

**Keywords:** neuroendocrine, tumor, PRRT, treatment, Canada

## Abstract

Pancreatic neuroendocrine tumors (pNETs) are rare but increasingly prevalent malignancies with varied prognoses and a diverse range of treatment options, including surgery, somatostatin analogues (SSAs), chemotherapy, targeted therapy, and peptide receptor radionuclide therapy (PRRT). This retrospective cohort study analyzed treatment patterns among 189 pNET patients treated between January 2010 and June 2021 at two Canadian cancer centres: the Verspeeten Family Cancer Centre (VFCC), which offers PRRT, and the Ottawa Hospital Cancer Centre (TOHCC), which does not at the time of the study. Data on demographics, tumor characteristics, and treatment modalities were collected, and statistical analyses were conducted using chi-square, Fisher’s exact test, and the Kruskal–Wallis test. Among eligible patients, 53% presented with stage IV disease. Surgical resection was the most common treatment, followed by SSAs, chemotherapy, PRRT, and targeted therapy. Stage IV patients at VFCC were significantly more likely to receive PRRT (60%) compared to TOHCC (6%) and underwent more PRRT cycles, with a higher prevalence of well-differentiated tumors observed at VFCC. With these differences it was clear that the non-PRRT centre was unable to provide patients with the same level of PRRT access during the study period compared to patients seen at the PRRT site. The findings underscore the critical role of PRRT availability in influencing treatment patterns and highlight the need for equitable access to specialized therapies across Canada to optimize outcomes for pNET patients.

## 1. Introduction

Neuroendocrine tumors (NETs) are a rare but rapidly growing class of neoplasms that are associated with varied survival rates depending on the primary tumor site, stage, and grade [1]. As per the SEER study in the period between 1973 and 2012, their incidence has increased 6.4-fold from 1.09 per 100,000 to 6.98 per 100,000. Of all the primary NET types, gastroenteropancreatic sites have the highest incidence rate, and pancreatic NETs (pNETs) specifically have the worst outcomes among the gastroenteropancreatic sites [1,2]. The median survival of a patient with a pNET is 5 years, but survival can range up to 50% at 20 years in localized, surgically resected disease [2]. This survival variance showcases the importance of early and appropriate treatment for favourable outcomes in pNET patients.

Treatment options for pNET include surgery; therapy, such as somatostatin analogues (SSAs); targeted therapy; chemotherapy; tumor ablation; and newer modalities, such as peptide receptor radionuclide therapy (PRRT) [3,4]. Surgical management remains the only curative treatment option for pNET patients and can also contribute to improved progression-free survival (PFS) and overall survival (OS) in some metastatic patients [5,6,7]. As OS can be very long in some patients, simple measures of life expectancy alone are not enough to determine the value provided to patients. Therefore, considering PFS is important in order to measure the quality of life patients experience. SSAs such as octreotide and lanreotide are the mainstays of the first-line management of NETs and have been in continued use since the late 1980s [8]. This class of medication works by binding to somatostatin receptors on NET cells to inhibit the secretion of hormones that contribute to the symptomatic load of cancer, such as diarrhea, flushing, and wheezing [9]. They can also be radiolabeled to be utilized as a diagnostic tool to identify disease presence and response to therapy [9].

Targeted therapy utilizes agents that specifically target NET cell development pathways. The currently approved targeted agents for pNETs are the mTOR inhibitor, everolimus, and the tyrosine kinase inhibitor, sunitinib [3]. These agents are generally reserved for later lines of treatment after progression on SSA therapy but can also be used as first-line therapy for pNETs, which do not express somatostatin receptors [3]. Other new multi-target tyrosine kinase inhibitors currently being used include cabozantinib and sulfatinib, but they currently require exceptional access programs [10].

Chemotherapy is also an option for patients who progress on SSAs and is used for palliation, prolonging survival, and in select cases, as neoadjuvant therapy [3]. The most frequent chemotherapy used in pNET is a combination of capecitabine and temozolomide; streptozotocin is used less frequently; platinum-based regimens are mainly employed in poorly differentiated carcinomas [3]. Chemotherapy for pNETs is still evolving, and the current focus is on identifying novel combined regimens for improved response rates to be used in patients who have failed other therapies [11].

PRRT is an evolution of the existing SSA treatment wherein a radioactive substance is linked to the SSA [12]. This allows for the delivery of radiation in a targeted manner to NETs expressing the appropriate somatostatin receptors (SSRs). The most used radioisotope is lutetium-177, which has a relatively short half-life and a favourable safety profile, especially for nephrotoxicity [12]. PRRT is extremely effective in NETs lower than grade 3, where the receptors are highly expressed in grade 3 NETs that express SSR [13]. However, the effectiveness drops once the Ki-67 value goes beyond 55% and somatostatin receptor expression decreases [13]. Studies are still identifying new targets for higher-grade tumors to utilize PRRT more effectively in these populations.

With a wide array of treatment options available, a multi-disciplinary approach involving surgeons, radiologists, and medical and radiation oncologists, among many others, is necessary. The choice of treatment will vary depending on a multitude of factors, including demographic factors, disease volume and distribution, available treatment resources, and expert consensus. This can lead to inter-centre variance in the treatment patterns of pNETs in different geographic regions. The increasing prevalence of NETs combined with their poor outcomes makes it an important area of research for improving the quality of care patients receive. The variability in the outcomes of pNETs make it important to identify treatment patterns. These current treatment patterns may aid clinicians in better understanding the present state of therapy to help guide the development of future guidelines.

Indeed, it should be noted that treatment patterns have recently changed. In 2022, the Canadian Agency for Drugs and Technologies in Health (CADTH) officially recommended funding for lutetium-based PRRT therapy for unresectable or metastatic pNETs [14]. Therefore, patients no longer need to be enrolled in special access programs to obtain access to this treatment modality in Canada. The main aim of this project was to identify the baseline treatment patterns of pNET patients, with a focus on PRRT prior to this funding change at two major Canadian centres, the Verspeeten Family Cancer Centre (VFCC) and the Ottawa Hospital Cancer Centre (TOHCC). These are, respectively, a PRRT treatment centre and a non-PRRT treatment centre.

## 2. Materials and Methods

This is a two-centre retrospective cohort study involving a PRRT (VFCC) and a non-PRRT treatment centre (TOHCC). It should be noted that TOHCC is currently a PRRT centre, but at the time of the study, it had yet to offer it. The study was approved by the research ethics boards of each institution. Patients over the age of 18 years with histologically confirmed pNETs and who were treated between January 2010 and June 2021 were identified using electronic health records. Patients were also required to have adequate kidney and liver function, as well as a good hematological profile of no more than 1.5× the institutional threshold.

Data collected included patient demographics (age and sex), geographic location (within or outside of a census metropolitan area per Statistics Canada) and distance to the PRRT centre, primary tumor (pancreas), and metastasis characteristics (location, differentiation, functionality, grade, presence of carcinoid syndrome, and data on the characteristics of treatment). A data dictionary was developed to ensure consistency across the centres. Study data were collected and managed using REDCap electronic data capture tools hosted at the Lawson Health Research Institute and TOHCC [15,16]. REDCap (Research Electronic Data Capture) is a secure, web-based software platform designed to support data capture for research studies, providing (1) an intuitive interface for validated data capture; (2) audit trails for tracking data manipulation and export procedures; (3) automated export procedures for seamless data downloads to common statistical packages; and (4) procedures for data integration and interoperability with external sources.

It should be noted that, in 2020, there was a change in practices at the VFCC as the medical oncologists saw the benefit of utilizing PRRT as the second line after progression on SSAs in GI NET and expanded the practice to pNETs as well rather than reserving it for later lines, as was carried out in previous years [17].

Statistical analysis: Categorical variables are presented as numbers with percentages and were compared using the chi-square or Fisher’s exact test. Continuous variables are presented as a median and range, and they were compared with the aid of the Kruskal–Wallis test. A *p*-value < 0.05 was considered statistically significant. All statistical analysis was performed using R software, version 4.2.1.

## 3. Results

In total, 189 pNET patients met the eligibility criteria, of which 42% were female and 40% were treated at the PRRT treatment centre (VFCC). The most common pNET locations were the pancreatic head (34%) and pancreatic tail (45%). Fifty-three percent (53%) of patients presented with stage IV disease, and twenty-one percent (21%) presented with stage II. Twenty-eight percent (28%) were grade 1, thirty-six percent (36%) were grade 2, and eight percent (8%) were grade 3, with the remainder either being unknown or having no pathology performed for the disease (Table 1).

There were no statistically significant differences in tumor characteristics when comparing male and female patients across both sites (Table 2).

In aggregate between the two sites, surgery remained the most common treatment modality used in the treatment of pNETs. SSAs were the most common non-surgical treatment, with 34% and 30% of patients receiving octreotide and lanreotide, respectively. Other treatments included chemotherapy, PRRT, and targeted therapy used in 30%, 29%, and 22% of patients, respectively. Most of the patients undergoing treatments other than primary surgery had metastatic, progressive disease (Table 3).

**Figure 1:**
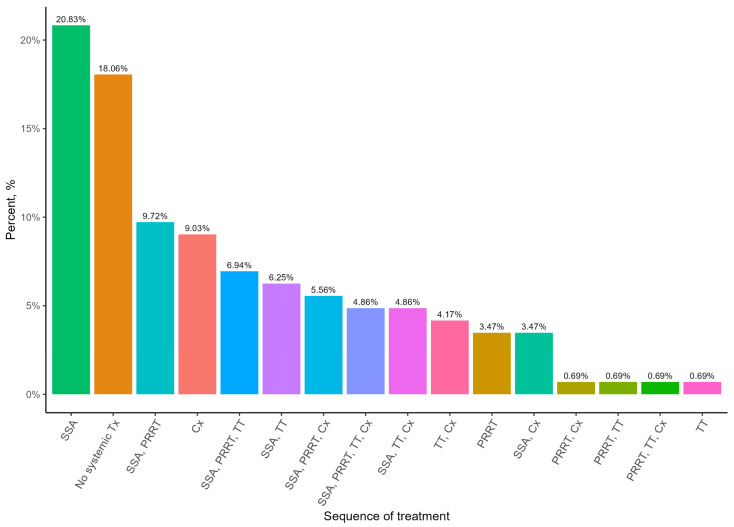
Most common sequences of non-surgical treatment for patients with Stage IV pNET. SSA—somatostatin analogue; TT—targeted therapy; Cx—chemotherapy.

When examining the treatment algorithms for pNET patients with stage IV disease, it appears that the most common treatment patients received was SSAs at 21%. A comparable amount of 18% underwent no systemic treatment. Chemotherapy was an important treatment option, with 9% of patients undergoing only chemotherapy. PRRT was most often used as a second-line treatment, with 25% of all patients following this pattern (Figure 1).

**Figure 2:**
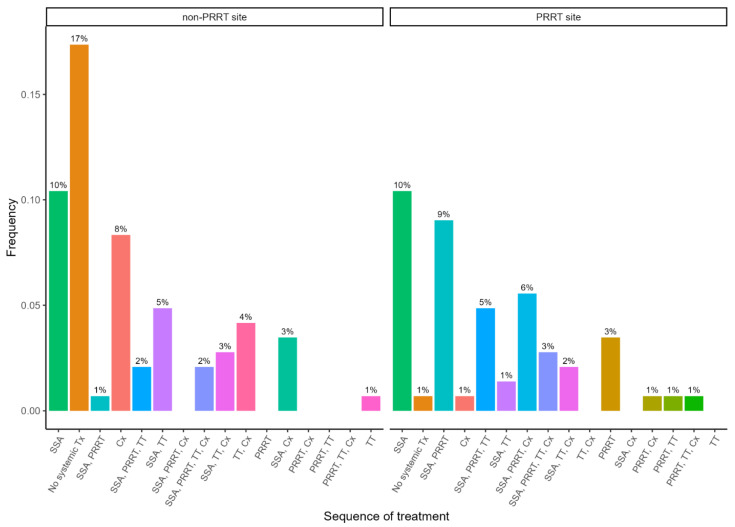
Most common sequences of non-surgical treatment by treatment site for Stage IV patients. SSA—somatostatin analogue; TT—targeted therapy; Cx—chemotherapy.

Comparing the two treatment sites, VFCC and TOHCC, patients received different sequences. SSA was used at the same rate at both sites; however, PRRT usage was much greater at the PRRT site, and chemotherapy was more utilized at the non-PRRT site. The non-PRRT site also had a higher number of patients for which no systemic treatments were used (Figure 2).

There were also some notable differences in the PRRT treatment demographics between the PRRT and non-PRRT centres. At the PRRT centre, more patients underwent PRRT, with 46 patients compared to the 7 patients at the non-PRRT centre. Eighty percent (80%) of PRRT patients at the PRRT centre had well-differentiated cancer compared to forty-three percent (43%) at the other site. The proportion of patients with stage IV pNETs treated with PRRT is nearly the same between the two sites. Surgery for the primary tumor was more common among patients at the PRRT centre. Both centres had a median of four cycles of PRRT, but the range of cycles was wider at the PRRT centre. Furthermore, a higher percentage of patients at the PRRT centre completed more than four rounds of PRRT treatment (Table 4).

The overall survival of PRRT and non-PRRT patients is comparable, but of the patients who died, non-PRRT patients had an increased likelihood of dying due to NET-related causes rather than PRRT patients (Table 5).

## 4. Discussion

In this real-world study, 53% of patients presented with non-curative NET disease. The most common systemic treatment used was SSAs, followed by chemotherapy, PRRT, and/or targeted therapy. Our results suggest that surgery for the primary tumor comprises the majority of the initial treatment. However, as most patients present with non-curative stage IV disease, systemic therapy and metastasectomy were necessary to achieve remission or palliation of the disease. From the data shown regarding treatment lines, it is evident that PRRT is an important second-line treatment for metastatic pNET patients. PRRT treatment has now become SOC based on two large randomized clinical trials. It is important that patients have access to all active treatments. In the setting where a treatment modality becomes centralized, it is important that barriers are addressed so that equal access is obtained. Our data did show, without allowing for a direct comparison due to the nature of retrospective data collection, that patients seen at a PRRT treatment centre did have a higher likelihood of being treated with PRRT.

However, even though PRRT was an important treatment line despite having similar numbers of pNET patients between the two sites, their usage of PRRT differed greatly. The PRRT centre treated 60% of patients with radionuclide therapy as opposed to only 6% at the non-PRRT centre. This study is the first Canadian study to look at PRRT treatment and distance to the PRRT centre during a time of special access request, and importantly, this observed difference in PRRT utilization suggests that distance to the treatment centre and other social reasons may impact the referral and use of PRRT. This difference is important; as specialized treatments become more centralized, we need to ensure that access is streamlined, or this may affect the uptake and use of novel therapies.

Additionally, patients from the non-PRRT centre who did receive PRRT did not complete as many cycles of therapy as the patients who received PRRT at the PRRT centre. Of the seven patients who underwent PRRT from the non-PRRT centre, three completed their treatment, and the rest discontinued due to the following: death (2/7), and (2/7) unknown reasons. As patients from the non-PRRT site had greater distances to travel to reach a treatment facility and had fewer cycles of treatment, the distance to the referral PRRT centre needs to be considered as an accessibility issue for patients. It could be that the logistics of organizing treatment over great distances impairs the ability and willingness of patients to complete therapy appropriately. However, it should be noted that this is a very small sample obtained from the non-PRRT site for drawing conclusions. Moreover, the simple fact that so few from the non-PRRT site were able to access PRRT treatment during the study period points to concerns about patient accessibility.

This question of access also leads to the limitations of this study. A significant factor affecting treatment selection is the simple availability and use of the treatment in question. In 2020, there was a change in practices at the VFCC as the medical oncologists saw the benefit of utilizing PRRT as the second line after progression on SSAs in GI NET and expanded the practice to pNETs rather than reserving it for later lines, as was carried out in previous years [17]. Thus, the PRRT centre began to utilize PRRT more based on this practice change. On the opposite side of the spectrum, for much of the study period, PRRT was only available at three treatment centres, which would mean at least a five-hour drive for many patients. Another limitation is the evaluation of outcomes based on biological sex. It has been established in other studies that females have increased survival from pNETs irrespective of the stage or morphology of the disease [18]. This study is unable to corroborate this due to the lack of sufficient statistical power to robustly analyze the survival difference and make any meaningful conclusion.

Our finding highlights the importance of ensuring regional access to new specialized programs such as RLT, or else this inherent bias may affect equal access to such programs. It has already been reported in a previous study by Hallet and colleagues that NET patients living in rural versus urban areas have increased cancer recurrences and decreased overall survival rates, which suggests another limitation for treatment availability [19].

## 5. Conclusions

This is one of the first cross-centre, patient-level Ontario studies that reports common treatment modalities used for the treatment of pNET. During a time of special access PRRT treatment for NET patients, the ease of accessing the program did affect the use of the treatment, with the PRRT site utilizing that treatment modality more. This highlights the importance of equitable access to this important treatment modality so that patients can receive the treatment that is most in keeping with the current literature on pNETs.

The incidence of this disease continues to rise in Ontario as in other jurisdictions around the world [1,20]. The treatment of this cancer will require more resources, especially as most present late with metastatic disease [20]. Therefore, it is important to investigate how treatment is being delivered and whether patients are receiving differential care based on access. This information will be useful to policymakers in determining resource allocation to ensure that patients are receiving the appropriate standard of care.

For the next steps, expanding the scope of our database to include other NET primary sites, such as the small bowel, lungs, and colon, will help further understand the current state of treatment and whether similarities or differences exist between various tumor sites.

## Figures and Tables

**Table 1:** Demographics and primary NET characteristics of patients reviewed at the PRRT and non-PRRT centre. Data from the time of diagnosis or first presentation to the cancer centre.

Demographic Information	PRRT Centre	Non-PRRT Centre
Biological sex (%)		
Female	39	43.5
Male	61	56.5
Median age at diagnosis [IQR]	61 [53, 66]	61 [54, 67]
Median height [Min, Max] (cm)	173 [151, 193]	168 [149, 188]
Median weight [Min, Max] (kg)	81 [45.0, 122]	81.5 [46.0, 148]
Living within a metropolitan area (%)	61	47.4
Median days from referral to medical oncology consultation	26	15
Median distance to PRRT centre (km)	112	569
**Clinical stage (%)**		
Stage I	13	20.9
Stage II	14.3	25.2
Stage III	10.4	7.8
Stage IV	62.3	46.1
Median Ki67 value	6	5
**Tumor grade (%)**		
Grade 1	27.9	27.2
Grade 2	44.1	30.4
Grade 3	5.9	9.8
Unknown	22.1	10.9

**Table 2:** Tumor characteristics separated by sex. *p*-value by Fisher’s exact test; Pearson’s chi-squared test.

Tumor Characteristic	Male (N = 112)	Female (N = 79)	*p*-Value
**Differentiation**			0.8
Well	66 (59%)	49 (62%)		
Moderate	2 (1.8%)	0 (0%)	
Poor	2 (1.8%)	1 (1.3%	
Unknown	42 (38%)	29 (37%)	
**Stage**			0.6
I	17 (15%)	17 (22%)	
II	23 (21%)	16 (20%)	
III	9 (8.0%)	8 (10%)	
IV	63 (56%)	38 (48%)	

**Table 3:** Summary of the treatment modalities used in aggregate between the PRRT and non-PRRT centres and the characteristics of the patients undergoing each therapy.

Treatment Modality	Patients Receiving Treatment (%)	Patient Characteristics
Surgery for Primary Tumor	56	40% curative intent
Octreotide	34	66% had progressive disease 82% had metastases at start
Lanreotide	30	77% had progressive disease 75% had metastases at start
Chemotherapy	30	94% had progressive disease and 91% had metastases at start 45% of treatments ceased due to disease progression 18% of treatments were completed
PRRT	29	Median number of cycles was 4 [1, 13] Of all treatment instances, 80% had progressive disease and 96% had metastases at treatment start 77% were completed
Targeted Therapy	22	85% had progressive disease 89% had metastases at treatment start
Surgery for Metastases	16	63% targeted the liver 31% had curative intent
Locoregional Ablative Therapy	16	99% of ablations targeted metastases
Radiation Therapy	15	93% of treatments were done with non-curative intent 100% had progressive disease 93% had metastases at treatment start
Lanreotide	30	77% had progressive disease 75% had metastases at start
Chemotherapy	30	94% had progressive disease and 91% had metastases at start 45% of treatments ceased due to disease progression 18% of treatments were completed

**Table 4:** Summary of the PRRT treatment characteristics between the PRRT and non-PRRT Centre. Completed treatment refers to treatment that was stopped by the clinician with a final note stating that the treatment course is complete without providing another reason for stopping therapy.

PRRT Treatment Characteristics	PRRT Centre	Non-PRRT Centre
Total number of pNET patients	77	112
Number of patients undergoing PRRT	46	7
Median distance to PRRT centre (km)	112 [IQR 34–193]	569 [IQR 551–593]
Median distance to PRRT centre if PRRT was used (km)	158 [IQR 85–541]	541 [IQR 155–576]
Patients undergoing PRRT with well-differentiated pNET (%)	80	43
Patients undergoing PRRT with stage IV pNET (%)	76	71
Surgery for primary tumor	74	54
Median number of cycles [Min, Max]	4 [1–13]	4 [1–5]

**Table 5:** Summary of pNET patient survival status.

pNET Patient Survival Status	PRRT	No PRRT
Alive (%)	47.2	52.9
Median Age at Diagnosis	58	62
Lost to Follow up (%)	1.9	2.9
Deceased (%)	50.9	44.2
Of Deceased NET-Related Death (%)	33.3	65.0

## Data Availability

The data that support the findings of this study are not publicly available because they contain protected patient health information (PHI). Non-PHI data are available upon reasonable request and with permission from the site investigators and the institutional research ethics board.
